# Hens and roosters of two distinct lines differ in immune cell profiles

**DOI:** 10.1016/j.psj.2026.106880

**Published:** 2026-03-27

**Authors:** Tanja Hofmann, Volker Stefanski, Werner Bessei, Sonja Schmucker

**Affiliations:** Institute of Animal Science, University of Hohenheim, Garbenstr. 17, 70599 Stuttgart, Germany

**Keywords:** Sexual dimorphism, Immune system, Chicken, Genetic line

## Abstract

Sexual dimorphism in immune function influences disease susceptibility, yet sex-specific immune characteristics in chickens remain poorly understood, particularly across genetically divergent lines. This study investigated sex- and line-dependent differences in the peripheral and lymphatic immune system in lines selected for high and low feather pecking. Immune cell subsets were quantified in blood, spleen and cecal tonsils, and functional immune parameters were analyzed by measuring plasma antibody concentration and mitogen-induced lymphocyte proliferation capacity. Distinct immunological differences were observed between roosters and hens, as well as between lines, with several effects being tissue- and sex-specific. The higher overall numbers of immune cells observed in the spleen of roosters were primarily attributable to their larger organ mass, whereas in the cecal tonsils this was mainly due to a higher cell density. Roosters had fewer peripheral B cells, lower antibody concentrations and B lymphocyte proliferation capacity, along with fewer CD4^+^ cells and a lower CD4^+^/CD8α^+^ ratio. However, they had more γδ T cells compared to hens, indicating a greater reliance on cytotoxic or innate immune mechanisms rather than on adaptive, B cell mediated immunity. Line-specific immune differences were also observed, with the number of CD4^+^ cells in the cecal tonsils being lower exclusively in high feather pecking hens, supporting the hypothesis that feather pecking might be an immune-related behavior, potentially based on immune imbalances in the gut. In conclusion, immune profiles are strongly influenced by both sex and genetic background. Our results further emphasize the importance of considering sex when analyzing immune responses, as well as in vaccination strategies, disease management, and breeding programs.

## Introduction

In many avian and mammalian species, sexual dimorphism in immune function plays a role in susceptibility to diseases. Often, females mount stronger immune responses to infections and vaccination than males, which can result in a faster clearance of pathogens, but also contributes to increased susceptibility to inflammatory and autoimmune diseases (Beagley and Gockel, 2003; Fish, 2008; Klein, 2012; Moulton, 2018; Shepherd et al., 2021). Sexual dimorphism in immune function is also known in other livestock species, including cattle (Burdick Sanchez et al., 2022; Carroll et al., 2015; Hulbert et al., 2013), pigs (Burdick Sanchez et al., 2017; Christoforidou et al., 2019; Fardisi et al., 2023) and poultry (Barbour et al., 1995; Leitner et al., 1989; Taylor et al., 1987; Rubel et al. 2025). Like mammals, female chickens show earlier, stronger and longer-lasting immune responses to vaccination (Barbour et al., 1995; Leitner et al., 1989), greater in vitro proliferation of T cells after stimulation with *E. coli* (Leitner et al., 1989), or higher phytohemagglutinin wattle response (Taylor et al., 1987) compared to male chickens. Hens also exhibit larger rectal mucosa-associated lymphoid tissue and higher local T-cell proliferation than roosters (Rubel et al., 2025). Consistent with these functional differences, recent single-cell transcriptomic analyses showed that male and female chickens differ in immune gene expression patterns, especially in B cells and proliferating T cells (Liu et al. 2026). Furthermore, roosters often exhibit more severe symptoms and higher mortality from infectious diseases (Barbour et al., 1995; Chacón et al., 2022; Leitner et al., 1989), though this pattern is not consistent across all pathogens or contexts (Boulton et al., 2018; Lipkin et al., 2022).

Beside the immunological parameters examined in the above-named studies (Barbour et al., 1995; Leitner et al., 1989; Liu et al. 2026; Rubel et al. 2025; Taylor et al., 1987,), no study has yet quantified sex differences in peripheral and intestinal immune cell numbers. Understanding sex-based immune dimorphism is essential. It enables the development of sex-targeted strategies to improve health, welfare, and resilience, by enabling more effective health management practices. These include optimized vaccination and therapeutic interventions, lowering disease burdens and pathogen persistence, thus leading ultimately to improved welfare, lower use of antibiotics, maximized productivity, reduced economic losses, and safer food products.

Moreover, different breeds of chickens display variations in immune function, driven by underlying genetic differences (Bereket Zekarias et al., 2002; Hofmann et al., 2025; Hofmann et al., 2021; Koenen et al., 2002; Schmucker et al., 2021). Over recent decades, selection criteria in layer lines have focused primarily on the hen, and male chicks were killed on the first day of life as they are not suitable for economically viable fattening. Recent studies have shown sex-dependent line differences in immunity, highlighting that genetic selection can affect roosters and hens differently (Lyte et al., 2025; Swaggerty, et al. 2024). However, sex-specific effects of genetic selection on immune function have largely been unexplored so far, although such differences could have profound consequences for disease susceptibility and health outcomes between roosters and hens. This knowledge gap becomes now relevant, as the killing of male chicks has been banned in Germany and France since 2022, and roosters of layer strains must be reared to slaughter age. Other EU countries are considering a similar approach (European Union, 2022). As a result, both sexes are exposed to production environments and health management strategies that have originally been optimized only for hens. Recently, differences in immune parameters were reported in hens selected for either high or low feather pecking behavior (Hofmann et al., 2025; van der Ejik et al., 2019). However, the selection for a specific trait may also result in sex-specific alterations in immune numbers.

Thus, this study aimed to investigate sex-specific differences between hens and roosters in numbers and function of leukocyte subsets of the systemic and lymphoid immune systems, and to determine whether these differences are influenced by genetic line resulting from selection for a single behavioral trait (high or low feather pecking).

## Materials and methods

All procedures were conducted in accordance with the relevant ethical and animal care guidelines and approved by the University of Hohenheim's Committee on Animal Care and Use (approval numbers T 223/23 and HOH 68/22-460f). All animals were hatched and raised at the Agricultural Experimental Station of the University of Hohenheim (Unterer Lindenhof, Eningen, Germany).

### Animals and housing

The experimental population derives from a White Leghorn layer strain, which was originally selected for high (**HFP**) and low feather pecking (**LFP**) behavior at the Danish Institute of Animal Science in Foulum, Denmark (for details, see Kjaer et al., 2001). After five generations of selection, hatching eggs from the HFP and LFP lines were relocated to the Research Station for Agricultural Science, at the University of Hohenheim, where the selection process based on severe feather pecking behavior (observed bouts per bird) of hens continued.

A total of 180 chickens, 120 hens (60 HFP, 60 LFP) and 60 roosters (30 HFP, 30 LFP), were randomly selected from the overall population of the 20^th^ generation. Based on binomial planning, 30 chickens per line and sex were required to achieve sufficient statistical power for analyzing immune differences. Because data from the hens were also used in a separate analysis that required a larger sample size, additional hens were included in this study. Chicks were hatched at the experimental station and tagged with a neck band that was fixed with plastic filaments injected under the skin, bearing a number for clear identification. From 8 weeks onwards, pullets and cockerls were raised separately under identical management practices (Lohmann Management Guide) within the same room. Each pen was furnished with wood shavings and provided a total usable area of 16.88 m² for 40 birds. Pens for hens and roosters were arranged on opposite sides of a central corridor. A consistent light schedule was maintained, with artificial lights switched on from 03:00 to 17:00 h, providing a basic light intensity of 20 lux. Windows were positioned in the upper sections of the roosters’ pens' side walls allowing additional natural light to enter, with intensities ranging from 20 to 2000 lux. Birds had free access to water and an antibiotic free diet, based on soy, corn and maize, that provided all recommended nutrients. It was mixed on site at the experimental station and was fed to both sexes. Birds received vaccinations against Marek’s disease via subcutaneous injection after hatching, as well as against coccidiosis, salmonellosis, Infectious Bronchitis, Newcastle Disease and Infectious Bursitis throughout the rearing period and against Newcastle Disease in the laying period at the experimental research center via drinking water.

### Sampling and sample preparation

At 42 to 43 wk of age, the chickens were slaughtered without prior feed withdrawal using CO₂ stunning, followed by a ventral neck cut for exsanguination. Slaughtering of all birds was conducted over 8 days within a 2-wk period due to technical constraints related to sample processing. All birds were slaughtered at the same time of day (morning hours) to minimize potential circadian effects on immune parameters. Hens and roosters were evenly distributed across the slaughter days, with each day including an equal number of birds of both sexes from each line.

Blood samples (4 mL) were collected in 5 mg/mL EDTA tubes (Sigma Aldrich, St. Louis, MO, USA). Samples were gently inverted 10 times to ensure proper mixing with the anticoagulant. Afterwards, 100 µL of blood was subsequently fixed for flow cytometry by TransFix® reagent (Caltag Medsystems Ltd., UK) in accordance with the manufacturer’s instructions and maintained at room temperature. The fixed and the remaining unfixed blood samples were further processed within 4 h of collection. Plasma samples for antibody analysis were obtained by the remaining unfixed blood sample by centrifuging unfixed blood samples for 15 min at 2000 × g and 4 °C and subsequently stored at -20 °C until measurement.

The spleen and both cecal tonsils were excised and preserved for transport on ice in PBS + 1 % Fetal Bovine Serum + 0.05 mg/mL Gentamycin (both Sigma Aldrich, St. Louis, MO, USA). The tissues were weighed prior to processing. Processing of lymphatic tissue followed the protocol outlined by Hofmann and Schmucker (2021). The spleen was aseptically sectioned into pieces and dissociated using a gentleMACS Dissociator (Miltenyi Biotec, Bergisch Gladbach, Germany). The resulting cell suspension was filtered (EasyStrainer, mesh size 40 μm; filter membrane polyethylene terephthalate; Greiner Bio-One, Kremsmünster, Austria) and the flow-through was centrifuged for 10 min at 300 × g at 4 °C. Intraepithelial lymphocytes of the CT were isolated from the mucosa via two cycles of tissue agitation under continuous rotation in Hanks’ Balanced Salt solution (without Mg^2+^ and Ca^2+^), supplemented by 5 mM EDTA (Sigma Aldrich, St. Louis, MO, USA), 5 % FBS (Sigma Aldrich, St. Louis, MO, USA), and 1 mM Dithiothreitol. The ensuing flow-through was centrifuged for 10 min at 300 × g at 20 °C. The cell pellets of spleen and CT were re-suspended, and the final volume was determined and stored on ice for subsequent processing.

### Flow cytometric analysis

Specific immune cell populations were characterized using flow cytometric analysis, followed by the no-lyse-no-wash method described by Seliger et al. (2012), with some modifications and amplifications, described in detail by Hofmann and Schmucker (2021) and Hofmann et al. (2025). Measurements were performed on a BD FACSCanto II (BD Biosciences, Heidelberg, Germany) equipped with a 488 nm blue laser, 630 nm red laser and a 405 nm violet laser. Acquisition and analysis were done using BD FACSDiva Software II (BD Biosciences, Heidelberg, Germany). The absolute number of leukocytes per µL blood or g tissue was determined using BD Trucount tubes (# 663028, BD Biosciences, Heidelberg, Germany) according to the manufacturer's instructions by dividing the number of cell events by the number of bead events. They were subsequently multiplied by bead concentration divided by sample volume per tube, recalculated based on sample dilution. Absolute cell numbers of leukocyte subsets were calculated by combining cell frequencies with total leukocyte counts. A minimum of 10,000 CD45^+^ cell events per blood sample, and of 50,000 CD45^+^ cell events per tissue sample, were collected. Cells were then stained for antibodies listed in Table 1. The anti-CD25 antibody was conjugated in-house to Alexa Fluor 647 (# A20186, Invitrogen/ThermoFisher Scientific, Waltham, MA, USA) following the manufacturer’s protocol. To consider autofluorescence and non-specific antibody binding of dead cells, SYTOX™ Blue Dead Cell Stain (# S34857, Thermo Fisher Scientific, Invitrogen, Waltham, MA) was used for exclusion of dead cells of the spleen and cecal tonsils. Unstained controls and isotype controls for CD45 (Table 1) were included for each individual bird to account for non-specific binding and autofluorescence, allowing accurate enumeration of total leukocytes. Corresponding gates were adjusted based on these controls to exclude background signals. After exclusion of cell doublets, cell events were gated based on the respective combination of surface marker expressions (see Appendix 1 for gating strategy).

**Table 1:** List of antibodies used for flow cytometry.

Antibody	Clone	Source	Identifier
Mouse Anti-Chicken CD45-APC	*LT40*	*SouthernBiotech*	*Cat# 8270-11, RRID:AB_2796475*
Mouse Anti-Chicken Monocyte/Macrophage-PE	Kul01	SouthernBiotech	Cat# 8420-09, RRID:AB_2796566
Mouse Anti-Chicken CD3-PE	CT-3	SouthernBiotech	Cat# 8200-09, RRID:AB_2796420
Mouse Anti-Chicken CD4-PACBLU	CT-4	SouthernBiotech	Cat# 8210-26, RRID:AB_2796434
Mouse Anti-Chicken CD4-PE-Cy-7	CT-4	SouthernBiotech	Cat# 8210-17, RRID:AB_2796433
Mouse Anti-Chicken CD4-PE	CT-4	SouthernBiotech	Cat# 8255-09, RRID:AB_2796466
Mouse Anti-Chicken CD8a-FITC	CT-8	SouthernBiotech	Cat# 8220-02, RRID:AB_2796439
Mouse Anti-Chicken Bu-1-FITC	AV20	SouthernBiotech	Cat# 8395-02, RRID:AB_2796542
Mouse Anti-Chicken CD41/CD61	11C3	Bio-Rad	Cat# MCA2240GA, RRID:AB_3100681
Mouse Anti-Chicken CD25	AV142	Bio-Rad	Cat# MCA5925GA, RRID:AB_3101160
Mouse Anti-Chicken TCR γδ (TCR1) - PerCP	TCR1	Novus	Cat# NBP1-28275PCP, RRID:AB_3204033
Mouse IgM-APC	1E1	SouthernBiotech	Cat# 0101-11, RRID:AB_2793838

Blood cells were classified in a single staining step as total leukocytes (CD45^+^), thrombocytes (CD45^dim^/CD41/61^+^), monocytes (CD45^+^/Kul01^+^), CD4^+^ cells (CD45^+^/CD4^+^/TCRγδ^−^), including T helper cells and regulatory T cells, CD8α^+^ cells (CD45^+^/CD4^−^/TCRγδ^−^/CD8α^+^), comprising cytotoxic T cells and natural killer cells, γδ T cells (CD45^+^/Kul01^−^/CD4^−^/TCRγδ^+^), and B cells (CD45^+^/Kul01^−^/Bu-1^+^). Heterophils were identified based on their FSC/SSC characteristics. Total lymphocyte counts were derived from the cumulative sum of CD4^+^ cells, CD8α^+^ cells, γδ T cells, and B cells.

In spleen and cecal tonsils, leukocyte subsets were stained in a total of three staining steps as total leukocytes (CD45^+^), thrombocytes (CD45^dim^/CD41/61^+^; only spleen), macrophages (CD45^+^/Kul01^+^; only spleen), T cells (CD45^+^/CD3^+^), natural killer cells (CD45^+^/CD3^-^/CD8α^+^), B cells (CD45^+^/Kul01^−^/Bu-1^+^), CD4^+^ cells (CD45^+^/CD3^+^/CD4^+^/TCRγδ^−^), including T helper cells and regulatory T cells, cytotoxic cells (CD45^+^/CD3^+^/CD4^−^/TCRγδ^−^/CD8α^+^), γδ T cells (CD45^+^/CD3^+^/CD4^−^/TCRγδ^+^), CD25^-^CD4^+^ cells, CD25^dim^CD4^+^ cells, and CD25^high^CD4^+^cells. Since upregulated expression of CD25 has been identified as a T cell activation marker in chickens (Hála et al., 1986; Naghizadeh et al., 2022), CD4^+^CD25d^im^ cells are likely activated T helper cells, while CD4^+^CD25^-^ cells may be resting T helper cells. Regulatory T cells are suggested to be primarily localized within the CD4^+^CD25^high^ population (Burkhardt et al., 2022).

### Splenic lymphocyte transformation assay

Activity of splenocytes was examined *in vitro* using a mitogen-induced lymphocyte proliferation assay employing ^3^H-thymidine incorporation as described in detail by Hofmann et al. (2021). In brief, isolated splenocytes were loaded on Biocoll (1.077 g/mL; Biochrom, Berlin, Germany) and centrifuged at 600 × g for 12 min at room temperature to obtain mononuclear cells. The interphase was collected, washed and re-suspended RPMI 1640 (Biochrom, Berlin, Germany) supplemented with 10 % FBS and 0.5 % Gentamycin (both Sigma Aldrich, St. Louis, MO, USA). Cell counts were determined using a Z2 Coulter Counter (Beckman Coulter, Krefeld, Germany). About 1.5 × 10^5^ cells per well were transferred into 96-well round bottom cell culture plates (Neolab, Heidelberg, Germany) in triplicate for each treatment. Cells were stimulated with either 10 μg/mL concanavalin A (ConA), or 10 μg/mL pokeweed mitogen (PWM) (Cat# C5275-5MG and Cat# L8777-5MG, respectively; both Sigma Aldrich, St. Louis, MO, USA) to stimulate T cells and T-cell-dependent B cell proliferation, respectively (Al-Khalifa, 2015), or left unstimulated as a negative control (medium only). The cells were then incubated at 41 °C in 5 % CO_2_ for 44 h. Subsequently, 0.25 μCi ^3^H-thymidine (PerkinElmer, Rodgau, Germany) per well was added for 24 h. The cells were harvested on glass fiber filters (Whatman, Buckinghamshire, United Kingdom), and the radioactivity incorporated was assessed in counts per minute (cpm) using a liquid scintillation analyzer (PerkinElmer, Rodgau, Germany). For each triplicate, mean of cpm was calculated and delta cpm for ConA and PWM generated (delta cpm = cpm of stimulated cells – cpm of unstimulated cells). CV of intra-assay for delta cpm of ConA was 11 % and for delta cpm of PWM 15 %.

### Enzyme-linked immunosorbent assay

Concentrations of IgY, IgM and IgA in plasma were determined by a Sandwich enzyme-linked immunosorbent assay (ELISA) as described in detail previously (Hofmann et al., 2021). The coating antibodies employed included goat anti-chicken IgY Fc antibody (# A30-104-A, 100 ng/well), goat anti-chicken IgM antibody (# A30-102-A, 200 ng/well), and goat anti-chicken IgA antibody (# A30-103-A, 200 ng/well) (all Bethyl Laboratories, Montgomery, TX, USA). Horseradish peroxidase-labeled goat anti-chicken IgY Fc (# A30-104-P, diluted 1:50,000), goat anti-chicken IgM (# A30-102-P, diluted 1:100,000), or goat anti-chicken IgA antibody (# A30-103-P, diluted 1:100,000) (all Bethyl Laboratories, Montgomery, TX, USA) were used as detection antibodies. Plasma samples were diluted 1:200,000, 1:10,000, 1:2,000 for IgY, IgM and IgA, respectively. All samples were quantified by reference to a calibration curve set up with a pooled plasma control whose IgY, IgM, and IgA concentrations were determined in advance with a Chicken IgG ELISA Kit (targeting chicken IgY), Chicken IgM ELISA Kit, and Chicken IgA ELISA Kit (all from Bethyl Laboratories, Montgomery, TX, USA). The calibration curve of IgY, IgM, and IgA ranged from 4.96 to 300 ng/mL, from 7.8 to 500 ng/mL, and from 15.63 to 1000 ng/mL, respectively. Absorbance was measured at 450 nm, and antibody concentration was calculated relative to the absorbance of the calibration curve. The coefficient of intra-assay variation was 4.04 % for IgY, 3.42 % for IgM, and 6.50 % for IgA. The coefficient of inter-assay variation was 12.02 % for IgY, 4.60 % for IgM, and 4.96 % for IgA.

### Statistical analysis

Statistical analysis was performed using a linear mixed model with the PROC MIXED procedure of the software package SAS (version 9.4; SAS Institute Inc., Cary, NC, USA), denoting *P* < 0.05 as being significant. Residuals were checked for normal distribution, and homogeneous error variance via graphical check of residual plots (Kozak and Piepho, 2018). In order to stabilize variance and meet the distributional assumptions, all variables were logarithmized prior to analysis. The linear mixed model included sex (rooster or hen), line (HFP or LFP) and their interactions as fixed effects, slaughter date and pen as random effects, and body weight and slaughter time as fixed covariates. Error effects were assumed to have heterogeneous variances, as Monday slaughtering days showed larger variances compared to other days. After finding significant effects via a global F-test (α = 0.05), adjusted means were compared with the Fisher’s LSD test. Adjusted means were back-transformed for presentation purposes. Standard errors were back-transformed using the Delta method. Degrees of freedom were determined by the method of Kenward-Roger, and variance components were estimated using the restricted maximum likelihood (REML) method. The results are presented as LSmeans with their SEM.

## Results

### Body weight and organ mass

**Fig. 1:**
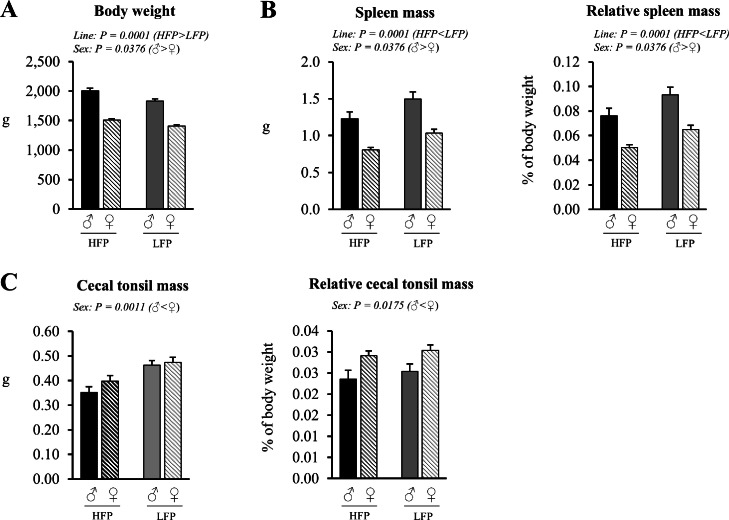
A Body weight of roosters (♂, *n* = 60) and hens (♀, *n* = 118) of a high (HFP) and low (LFP) feather pecking line (*n* = 89/line). B Absolute and relative spleen mass of roosters (♂, *n* = 59) and hens (♀, *n* = 117) of a high (HFP) and low (LFP) feather pecking line (*n* = 88/line). C Absolute and relative mass of cecal tonsils of roosters (♂, *n* = 58) and hens (♀, *n* = 111) of a high (HFP, *n* = 82) and low (LFP, *n* = 87) feather pecking line. Data is presented as LSmean ± SEM. Results of statistical analysis of the main effects (line and sex), as well as their interaction (line × sex), are given above each figure when *P* < 0.05. The directions of main effects are shown. In case of interaction effects, means that do not share a common lowercase letter differ at *P* < 0.05.

No significant sex × line interactions on body or organ mass were observed (Fig. 1). Roosters had a higher body weight (*P* < 0.0001), higher absolute and relative spleen mass (*P* < 0.0001), but lower absolute (*P* = 0.0011) and relative (*P* = 0.0175) mass of cecal tonsils compared to hens. HFP chickens had a higher body weight (*P* < 0.0001) and lower absolute and relative spleen mass (*P* < 0.0001) compared to LFP chickens, whereas both lines had a similar absolute and relative cecal tonsils mass (*P* = 0.0851 and *P* = 0.2786, respectively).

### Number and proportion of immune cells in blood, spleen and cecal tonsils

**Fig. 2:**
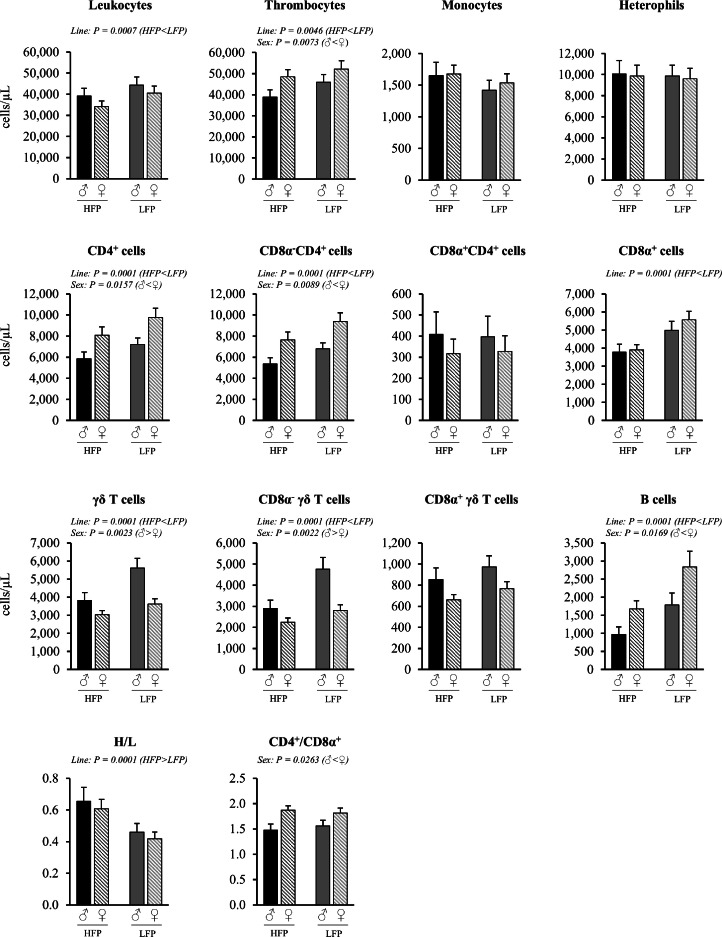
Immune cell numbers per µL blood, heterophil to lymphocyte ratio (H/L) and CD4^+^ cells to CD8α^+^ cell ratio in roosters (♂, *n* = 60) and hens (♀, *n* = 118) of a high (HFP, *n* = 89) and low (LFP, *n* = 89) feather pecking line. Data is presented as LSmean ± SEM. Results of statistical analysis of the main effects (line and sex), as well as their interaction (line × sex), are given above each figure when *P* < 0.05. The directions of main effects are shown. In case of interaction effects, means that do not share a common lowercase letter differ at *P* < 0.05.

**Fig. 3:**
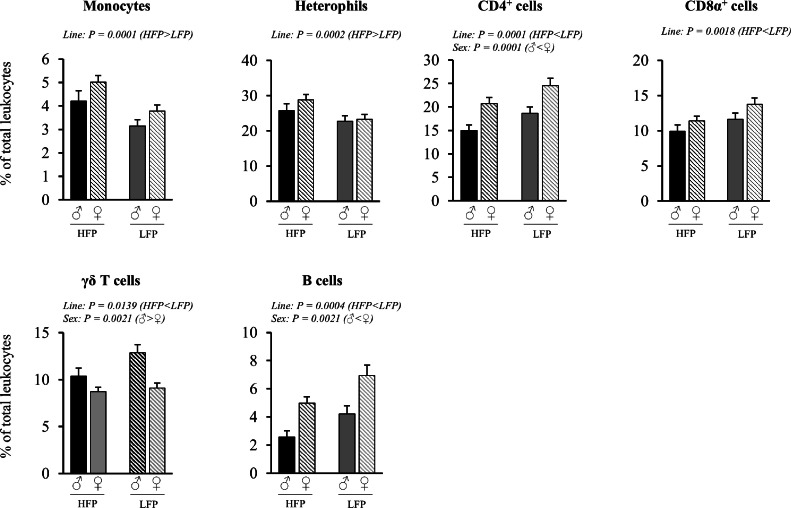
Relative proportions of leukocyte subsets in blood in roosters (♂, *n* = 60) and hens (♀, *n* = 118) of a high (HFP, *n* = 89) and low (LFP, *n* = 89) feather pecking line. Data is presented as LSmean ± SEM. Results of statistical analysis of the main effects (line and sex), as well as their interaction (line × sex), are given above each figure when *P* < 0.05. The directions of main effects are shown. In case of interaction effects, means that do not share a common lowercase letter differ at *P* < 0.05.

**Fig. 4:**
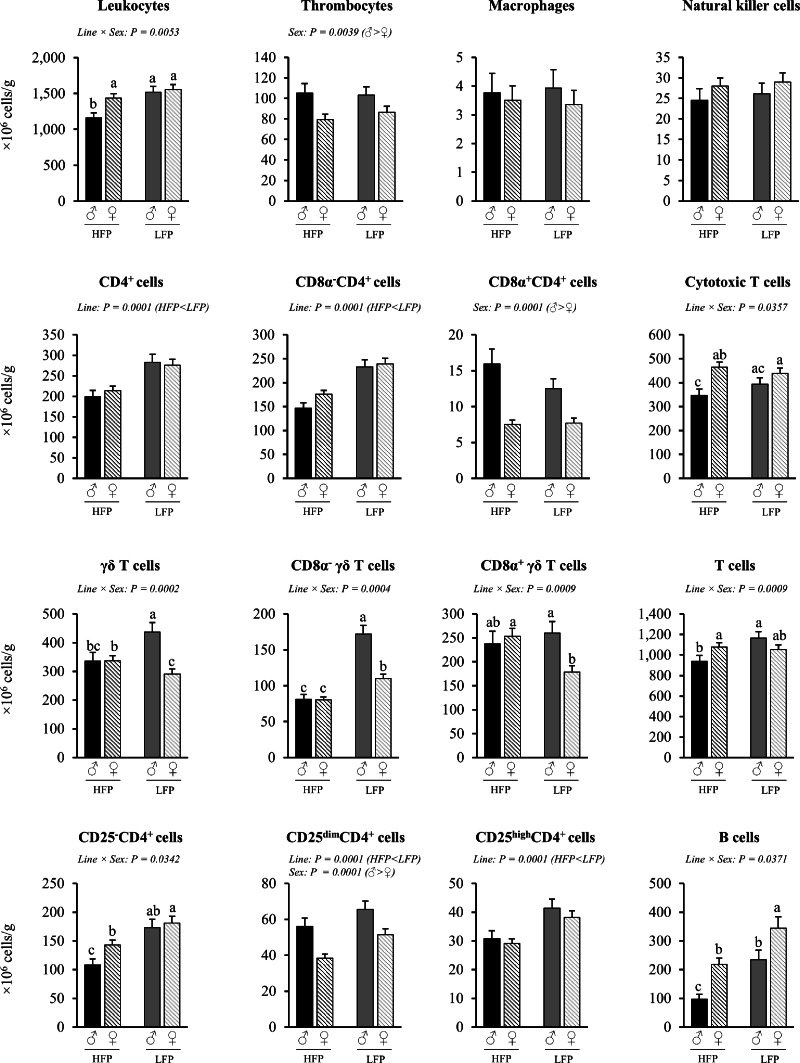
Immune cell numbers per g spleen in roosters (♂, *n* = 59) and hens (♀, *n* = 117) of a high (HFP, *n* = 88) and low (LFP, *n* = 88) feather pecking line. Data is presented as LSmean ± SEM. Results of statistical analysis of the main effects (line and sex), as well as their interaction (line × sex), are given above each figure when *P* < 0.05. The directions of main effects are shown. In case of interaction effects, means that do not share a common lowercase letter differ at *P* < 0.05.

**Fig. 5:**
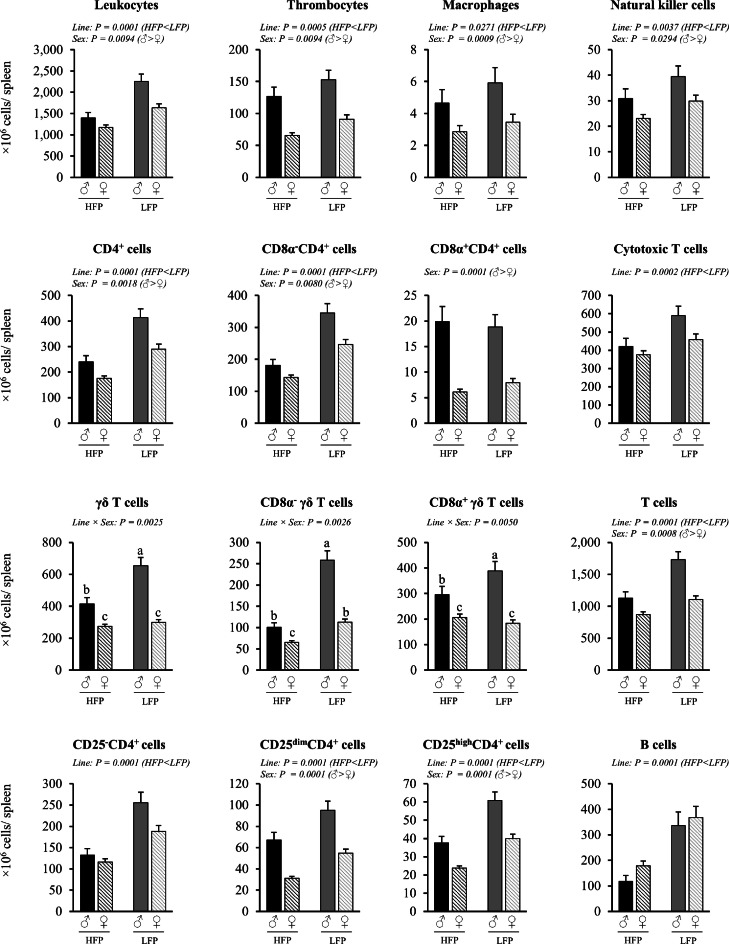
Immune cell numbers per spleen in roosters (♂, *n* = 59) and hens (♀, *n* = 117) of a high (HFP, *n* = 88) and low (LFP, *n* = 88) feather pecking line. Data is presented as LSmean ± SEM. Results of statistical analysis of the main effects (line and sex), as well as their interaction (line × sex), are given above each figure when *P* < 0.05. The directions of main effects are shown. In case of interaction effects, means that do not share a common lowercase letter differ at *P* < 0.05.

**Fig. 6:**
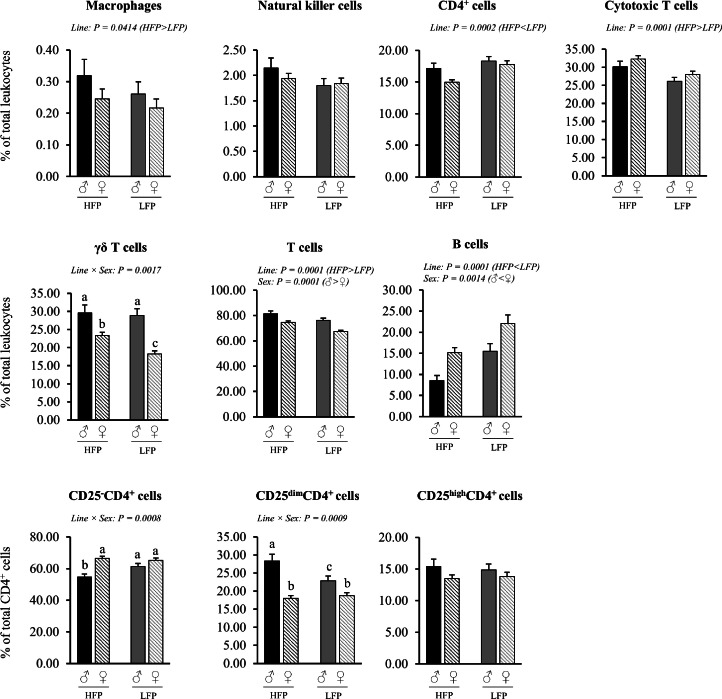
Relative proportions of leukocytes subsets in total spleen in roosters (♂, *n* = 59) and hens (♀, *n* = 117) of a high (HFP, *n* = 88) and low (LFP, *n* = 88) feather pecking line. Data is presented as LSmean ± SEM. Results of statistical analysis of the main effects (line and sex), as well as their interaction (line × sex), are given above each figure when *P* < 0.05. The directions of main effects are shown. In case of interaction effects, means that do not share a common lowercase letter differ at *P* < 0.05.

**Fig. 7:**
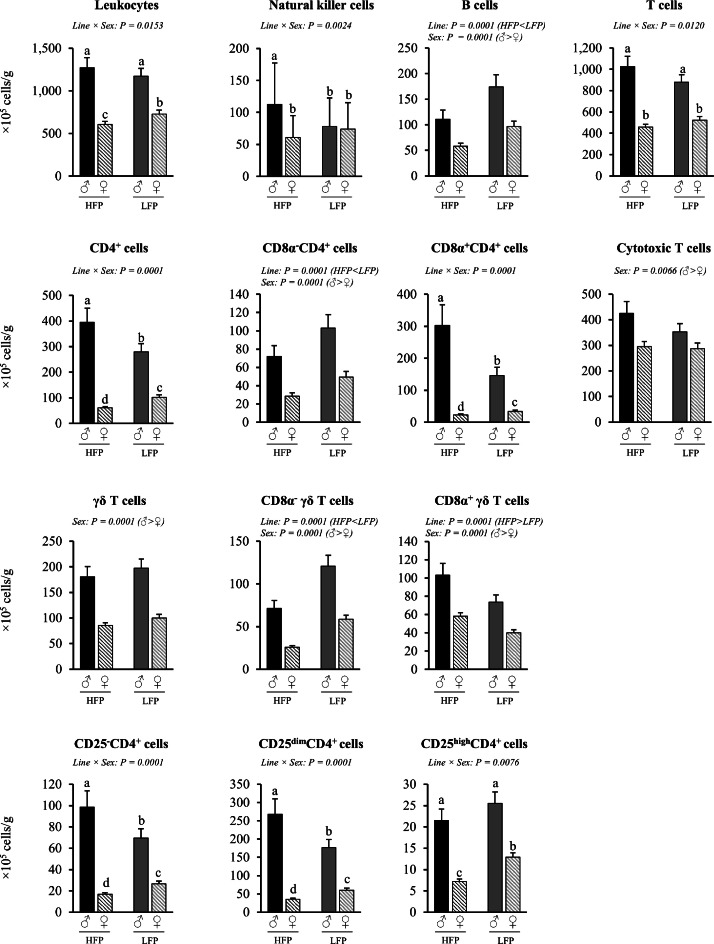
Immune cell numbers per g cecal tonsils in roosters (♂, *n* = 58) and hens (♀, *n* = 111) of a high (HFP, *n* = 82) and low (LFP, *n* = 87) feather pecking line. Data is presented as LSmean ± SEM. Results of statistical analysis of the main effects (line and sex), as well as their interaction (line × sex), are given above each figure when *P* < 0.05. The directions of main effects are shown. In case of interaction effects, means that do not share a common lowercase letter differ at *P* < 0.05.

**Fig. 8:**
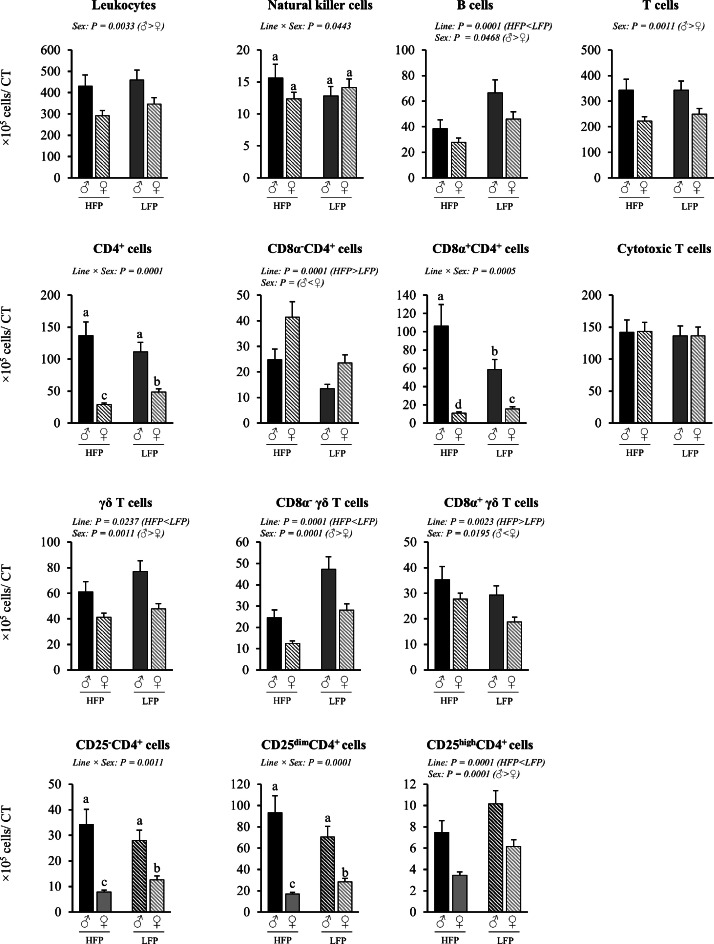
Immune cell numbers in entire cecal tonsils in roosters (♂, *n* = 58) and hens (♀, *n* = 111) of a high (HFP, *n* = 82) and low (LFP, *n* = 87) feather pecking line. Data is presented as LSmean ± SEM. Results of statistical analysis of the main effects (line and sex), as well as their interaction (line × sex), are given above each figure when *P* < 0.05. The directions of main effects are shown. In case of interaction effects, means that do not share a common lowercase letter differ at *P* < 0.05.

**Fig. 9:**
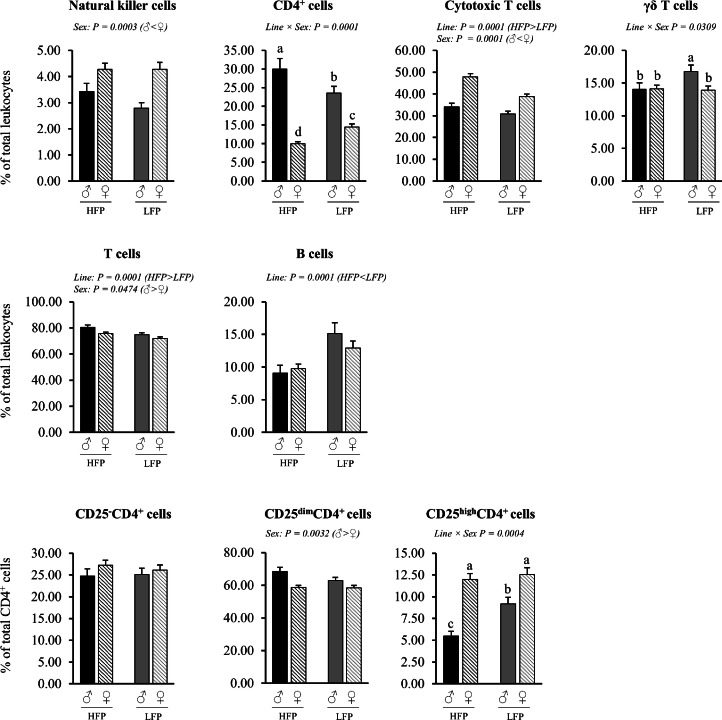
Relative proportion of leukocyte subsets among total leukocytes in cecal tonsils in roosters (♂, *n* = 58) and hens (♀, *n* = 111) of a high (HFP, *n* = 82) and low (LFP, *n* = 87) feather pecking line. Data is presented as LSmean ± SEM. Results of statistical analysis of the main effects (line and sex), as well as their interaction (line × sex), are given above each figure when *P* < 0.05. The directions of main effects are shown. In case of interaction effects, means that do not share a common lowercase letter differ at *P* < 0.05.

Cells per µL blood or gram tissue were quantified in order to assess the relative cell density independent of organ or body mass. The total numbers of immune cells per organ (spleen, cecal tonsils) were determined to assess the overall immune cell reservoir, and the relative proportions of leukocyte subsets were assessed to evaluate shifts in immune composition and balance. Mixed linear model analysis revealed significant interaction effects of sex × line (*P* < 0.05) in both lymphatic tissues, as well as significant effects for the number and proportion of immune cells of sex (*P* < 0.05) and line (*P* < 0.05) in blood (Fig. 2, Fig. 3), spleen (Fig. 4, Fig. 5, Fig. 6), and cecal tonsils (Fig. 7, Fig. 8, Fig. 9).

***Blood (cells/µL).*** No significant sex × line interactions were observed in the number of immune cells per µL blood (Fig. 2). Irrespective of line, roosters had lower numbers of thrombocytes (*P* = 0.0073), total CD4^+^ cells (*P* = 0.0157), and B cells (*P* = 0.0169), but higher numbers of γδ T cells (*P* = 0.0023) per µL blood compared to hens. The numbers of leukocytes (*P* = 0.0875), heterophils (*P* = 0.6563), monocytes (*P* = 0.6880) and total CD8α^+^ cells (*P* = 0.5252) were similar between roosters and hens. The ratio of heterophils to lymphocytes (H/L) was similar in both sexes (*P* = 0.4583), while the ratio of CD4^+^ to CD8α^+^ cells (CD4^+^/CD8α^+^) was lower in roosters compared to hens (*P* = 0.0263). Irrespective of sex, HFP chickens had lower numbers of total leukocytes (*P* = 0.0007), thrombocytes (*P* = 0.0046), total CD4^+^ cells (*P* = 0.0001), total CD8α^+^ cells (*P* = 0.0001), γδ T cells (*P* = 0.0001), and B cells (*P* = 0.0001) per µl blood compared to LFP chickens. The numbers of peripheral heterophils (*P* = 0.9223) and monocytes (*P* = 0.1332) did not differ between the two lines. HFP chickens showed a higher H/L ratio (*P* = 0.0001) than LFP chickens, while the CD4^+^/CD8α^+^ ratio (*P* = 0.8237) was comparable between both lines.

***Blood (% of total leukocytes).*** No significant sex × line interactions were observed for the relative proportion of leukocyte subsets in blood (Fig. 3). Irrespective of line, roosters had lower proportions of CD4^+^ cells (*P* = 0.0001) and B cells (*P* = 0.0021), but higher proportions of γδ T cells among leukocytes (*P* = 0.0021) compared to hens. Irrespective of sex, HFP chickens had higher proportions of monocytes (*P* = 0.0001) and heterophils (*P* = 0.0002), but lower proportions of total CD4^+^ cells (*P* = 0.0001), total CD8α^+^ cells (*P* = 0.0018), γδ T cells (*P* = 0.0021) and B cells (*P* = 0.0021) among leukocytes compared to LFP chickens.

***Spleen (cells/g).*** Significant interaction effects of sex × line were found for the number of leukocytes (*P* = 0.0053), total T cells (*P* = 0.0009), cytotoxic T cells (*P* = 0.0357), γδ T cells (*P* = 0.0002), B cells (*P* = 0.0371), and CD25^-^CD4^+^ cells (*P* = 0.0342) per g spleen (Fig. 4). Within the HFP line, roosters had lower numbers of total leukocytes (*P* = 0.0019), total T cells (*P* = 0.0483), cytotoxic T cells (*P* = 0.0012), and CD25^-^CD4^+^cells (*P* = 0.0140) per g spleen compared to hens. In contrast, within the LFP line, no differences in the number of cells of the respective cell types per g spleen were observed between sexes (*P* = 0.7090, *P* = 0.1206; *P* = 0.2097, and *P* = 0.6932, respectively). However, within the LFP line, roosters had higher numbers of γδ T cells (*P* = 0.0001) per g spleen than hens, whereas no difference was found for the numbers of γδ T cells (*P* = 0.9713) between roosters and hens of the HFP line. This effect was also found for CD8α^-^γδ T cells (LFP ♂ vs. ♀: *P* = 0.0001; HFP ♂ vs. ♀: *P* = 0.9675) and CD8α^+^γδ T cells (LFP ♂ vs. ♀: *P* = 0.0031; HFP ♂ vs. ♀: P=0.6279). Regarding line effects within the sexes, roosters of the HFP line had lower numbers of total leukocytes (*P* = 0.0001) and total T cells (*P* = 0.0002) per g spleen than roosters of the LFP line, whereas no differences were found for these cell types between the hens of the two lines. Moreover, roosters of the HFP line also had lower numbers of γδ T cells compared to roosters of the LFP line (*P* = 0.0042), whereas the opposite result was found in hens, with higher numbers in hens of the HFP line compared to hens of the LFP line (*P* = 0.0223). The number of B cells was lower in HFP chickens compared to LFP chickens of both sexes (♂ HFP vs. LFP: *P* = 0.0001; ♀ HFP vs. LFP: *P* = 0.0001). In both lines, roosters had lower numbers of B cells compared to hens (HFP ♂ vs. ♀: *P* = 0.0001; LFP ♂ vs. ♀: *P* = 0.0479).

Irrespective of line, roosters had higher numbers of thrombocytes (*P* = 0.0039), CD8α^+^ CD4^+^ cells (*P* = 0.0001) and CD25^dim^CD4^+^ cells (*P* = 0.0001) than hens. Irrespective of sex, HFP chickens had lower numbers of total CD4^+^ cells (*P* = 0.0001), CD8α^-^CD4^+^ cells (P 0 0.0001), CD25^dim^CD4^+^ cells (*P* = 0.0001) and CD25^high^CD4^+^ cells (*P* = 0.0001) compared to LFP chickens.

***Spleen (cells/spleen).*** A significant interaction effect of sex × line was found for the number of γδ T cells (*P* = 0.0025) per spleen (Fig. 5). Roosters of the HFP line had fewer γδ T cells per spleen than roosters of the LFP line (*P* = 0.0001), whereas this effect was not observed for the hens (*P* = 0.2081). Similar effects were also found for CD8α^-^ and CD8α^+^γδ T cells. In addition, the number of total γδ T cells per spleen was higher in roosters than in hens (HFP ♂ vs. ♀: *P* = 0.0005; LFP ♂ vs. ♀: *P* = 0.0001).

Irrespective of line, roosters had overall higher numbers of leukocytes (*P* = 0.0094), thrombocytes (*P* < 0.0001), macrophages (*P* = 0.0009), natural killer cells (*P* = 0.0294), total T cells (*P* = 0.0001), total CD4^+^ cells (*P* = 0.0018), CD25^dim^CD4^+^ cells (*P* < 0.0001), and CD25^high^CD4^+^ cells (*P* < 0.0001) per spleen compared to hens. Irrespective of sex, HFP chickens had lower numbers of leukocytes (*P* < 0.0001), thrombocytes (P= 0.0005), macrophages (*P* = 0.0271), natural killer cells (*P* = 0.0037), total T cells (*P* < 0.0001), total CD4^+^ cells (*P* < 0.0001), cytotoxic T cells (*P* = 0.0002), B cells (*P* < 0.0001), CD25^-^CD4^+^ cells (*P* < 0.0001), CD25^dim^CD4^+^ cells (*P* < 0.0001), and CD25^high^CD4^+^ cells (*P* < 0.0001) per spleen than LFP chickens.

Spleen (% of total leukocytes). Significant interaction effects of sex × line were found for the relative proportion of γδ T cells (*P* = 0.0017), CD25^-^CD4^+^ cells (*P* = 0.007) and CD25^dim^CD4^+^ cells (*P* = 0.008) (Fig. 6). In detail, hens of the HFP line had higher proportions of γδ T cells among leukocytes than hens of the LFP line (*P* = 0.0001), whereas no line effect was observed in the roosters (*P* = 0.7824). In addition, roosters of the HFP line had lower proportions of CD25^-^CD4^+^ cells (*P* = 0.0007), but higher proportions of CD25^dim^CD4^+^ cells than roosters of the LFP line (*P* = 0.0010), while no such effects were observed in the hens (*P* = 0.3908 and *P* = 0.3674, respectively). Within the HFP line, roosters had lower proportions of CD25^-^CD4^+^ cells compared to hens (*P* = 0.0001), while no sex effects were observed within the LFP line (*P* = 0.1344). Across both lines, roosters had higher proportions of γδ T cells (HFP ♂ vs. ♀: *P* = 0.0116; LFP ♂ vs. ♀: *P* < 0.0001), and CD25^dim^CD4^+^cells (HFP ♂ vs. ♀: *P* < 0.0001; LFP ♂ vs. ♀: *P* = 0.0001) compared to hens.

Irrespective of line, roosters had higher proportions of total T cells (*P* = 0.0001) and lower proportions of B cells (*P* = 0.0014) among leukocytes in the spleen compared to hens. Irrespective of sex, HFP chickens had higher proportions of macrophages (*P* = 0.0414), total T cells (*P* = 0.0001), and cytotoxic T cells (*P* = 0.0001), but lower proportions of B cells (*P* = 0.0014) compared to LFP chickens.

Cecal Tonsils (cells/g). Significant interaction effects of sex × line were found for the number of leukocytes (*P* = 0.0153), natural killer cells (*P* = 0.0024), T cells (*P* = 0.012), total CD4^+^ cells (*P* = 0.0001), CD8α^+^CD4^+^ cells, CD25^-^CD4^+^cells (*P* = 0.0001), CD25^dim^CD4^+^ cells (*P* = 0.0001), and CD25^high^CD4^+^ cells (*P* = 0.0076) per g cecal tonsils (Fig. 7). In detail, hens of the HFP line had lower numbers of leukocytes (*P* = 0.0055) and CD25^high^CD4^+^ cells (*P* = 0.0001) compared to hens of the LFP line, whereas these line effects were not observed in roosters (*P* = 0.3698 and *P* = 0.1738, respectively). In contrast, roosters of the HFP line had higher numbers of natural killer cells (*P* = 0.0177) compared to roosters of the LFP line, while no effect was observed in hens (*P* = 0.0735). Interestingly, roosters of the HFP line had higher numbers of total CD4^+^ cells (*P* = 0.0178), CD8α^+^ CD4^+^ cells (*P* = 0.0012), CD25^-^CD4^+^ cells (*P* = 0.0316), and CD25^dim^CD4^+^ cells (*P* = 0.0100) per g cecal tonsils compared to roosters of the LFP line. In contrast, the opposite pattern was observed in hens, with those from the LFP line having higher numbers of the respective CD4^+^ cell subsets than hens from the HFP line (*P* = 0.0001, *P* = 0.0211, *P* = 0.0001, and *P* = 0.0001, respectively). Within each line, roosters had higher numbers of leukocytes, total T cells, total CD4^+^ cells, CD8α^+^CD4^+^ cells, as well as CD25^-^CD4^+^ cells, CD25^dim^CD4^+^ cells, and CD25^high^CD4^+^ cells than hens (♂ HFP vs. ♀ HFP, ♂ HLFP vs. ♀ LFP, *P* = 0.0001 for all cell types). In addition, a higher number of natural killer cells was observed in roosters compared to hens in the HFP line (*P* = 0.0012), whereas no such difference was found in the LFP line (*P* = 0.7455).

Irrespective of line, roosters had higher numbers of B cells (*P* = 0.0001), cytotoxic T cells (*P* = 0.0066), and γδ T cells (*P* < 0.0001) compared to hens. Irrespective of sex, HFP chickens had lower numbers of B cells (*P* < 0.0001) compared to LFP chickens.

Cecal Tonsils (cells/cecal tonsils). Significant interaction effects of sex × line were found for the number of natural killer cells (*P* = 0.0443), total CD4^+^ cells (*P* = 0.0001), CD8α^+^CD4^+^ cells (*P* = 0.0005), as well as CD25^-^CD4^+^ cells (*P* = 0.0011) and CD25^dim^CD4^+^ cells (*P* = 0.0001) in the entire cecal tonsil (Fig. 8). Within both lines, roosters had higher numbers of total CD4^+^ cells (HFP ♂ vs. ♀: *P* = 0.0001; LFP ♂ vs. ♀: *P* = 0.0001), CD8α^+^CD4^+^ cells (HFP ♂ vs. ♀: *P* = 0.0013; LFP ♂ vs. ♀: *P* = 0.0018), CD25^-^CD4^+^ cells (HFP ♂ vs. ♀: *P* = 0.0001; LFP ♂ vs. ♀: *P* = 0.0001), and CD25^dim^CD4^+^ cells (HFP ♂ vs. ♀: *P* = 0.0001; LFP ♂ vs. ♀: *P* = 0.0001), but with a higher effect between roosters and hens within the HFP line. Regarding line effects within sexes, hens of the HFP line had lower numbers of total CD4^+^ cells (*P* = 0.0001), CD25^-^CD4^+^ cells (*P* = 0.0002), and CD25^dim^CD4^+^ cells (*P* = 0.0001) compared to hens of the LFP line. In contrast, no effect of line was observed for roosters for the respective immune cell types (*P* = 0.1834, *P* = 0.2433, and *P* = 0.0966, respectively). The number of CD8α^+^CD4^+^ cells was lower in hens of the HFP line compared to hens of the LFP line (*P* = 0.0304), whereas the opposite was observed in roosters, with higher numbers in the HFP line (*P* = 0.0091).

Irrespective of line, roosters had higher numbers of leukocytes (*P* = 0.0033), B cells (*P* = 0.0468), total T cells (*P* = 0.0011), γδ T cells (*P* = 0.0011), and CD25^high^CD4^+^ cells (*P* = 0.0001) in the entire cecal tonsil compared to hens. Irrespective of sex, HFP chickens had lower numbers of B cells (*P* = 0.0001), γδ T cells (P= 0.0237), and CD25^high^CD4^+^ cells (*P* = 0.0001) compared to LFP chickens.

Cecal Tonsils (% of total leukocytes). Significant interaction effects of sex × line were found for the relative proportion of total CD4^+^ cells (*P* = 0.001), γδ T cells (*P* = 0.0309), and CD25^high^CD4^+^ cells (*P* = 0.0004) among leukocytes (Fig. 9). Within both lines, roosters had higher proportions of total CD4^+^ cells (HFP/LFP ♂ vs. ♀: both *P* = 0.0001), but lower proportions of CD25^high^CD4^+^ cells (HFP ♂ vs. ♀: *P* = 0.0001; LFP ♂ vs. ♀: *P* = 0.0120) compared to hens, again with a greater effect within the HFP line. Roosters of the LFP line had higher proportions of γδ T cells compared to hens of the LFP line (*P* = 0.0268), whereas no sex effect was observed within the HFP line (*P* = 0.9740). Regarding line effects within sexes, roosters of the HFP line had a higher proportion of total CD4^+^ cells (*P* = 0.0144), while the opposite was observed for hens (*P* = 0.0001). Regarding sex effects within lines, roosters of the HFP line had higher proportions of γδ T cells and lower proportions of CD25^high^CD4^+^ cells (*P* = 0.0001) compared to roosters of the LFP line (*P* = 0.0176), while no line effect was observed within the hens (*P* = 0.8041 and *P* = 0.5714, respectively).

Irrespective of line, roosters had a lower proportion of natural killer cells (*P* = 0.0003) and cytotoxic T cells (*P* = 0.0001), but higher proportions of total T cells (*P* = 0.0474) and CD25^dim^CD4^+^ cells (*P* = 0.0032) in the cecal tonsils compared to hens. Irrespective of sex, HFP chickens had lower proportions of B cells (*P* = 0.0001) compared to LFP chickens.

### Functionality of immune cells

**Fig. 10:**
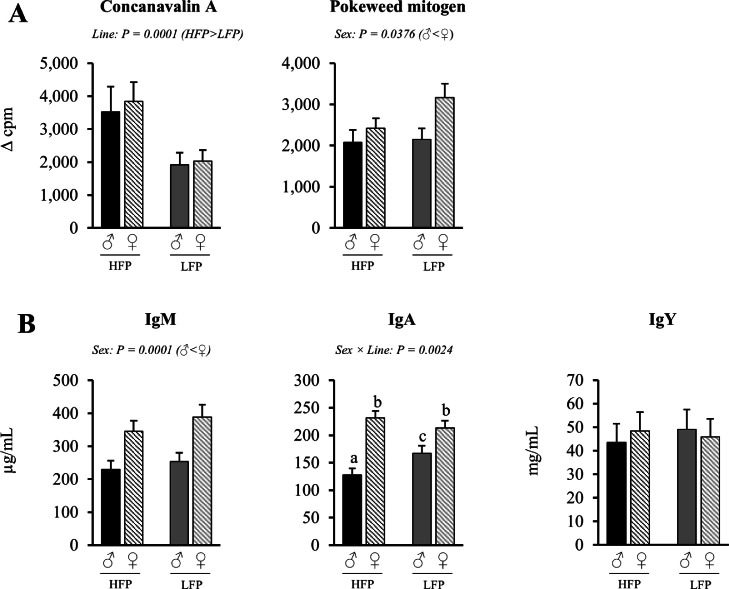
A Mitogen-induced splenic lymphocyte proliferation capacity after stimulation with ConcavanalinA and Pokeweed Mitogen in hens (♀, *n* = 117 and *n* = 101) and roosters (♂, *n* = 60 and *n* = 51) of a high (HFP, *n* = 88 and *n* = 75) and low (LFP, *n* = 89 and *n* = 77) feather pecking line. B Antibody concentration in plasma of hens (♀, *n* = 119) and roosters (♂, *n* = 60) of a high (HFP, *n* = 90) and low (LFP, *n* = 89) feather pecking line. Data is presented as LSmean ± SEM. Results of statistical analysis of the main effects (line and sex), as well as their interaction (line × sex), are given above each figure when *P* < 0.05. The directions of main effects are shown. In case of interaction effects, means that do not share a common lowercase letter differ at *P* < 0.05.

The mixed linear model analysis revealed significant effects for sex (*P* < 0.05) and line (*P* < 0.05) for splenic lymphocyte proliferation capacity (Fig. 10A) and plasma IgM antibody levels (Fig. 10B), as well as a significant interaction effect of sex × line (*P* < 0.05) for IgA antibody levels in plasma.

### Lymphocyte proliferation capacity of splenic lymphocytes

Roosters exhibited overall lower PWM-induced lymphocyte proliferation compared to hens (*P* = 0.0376), while ConA-induced lymphocyte proliferation was similar between sexes (*P* = 0.7142) (Fig. 10A). HFP chickens exhibited higher ConA-induced lymphocyte proliferation (*P* = 0.0001) compared to LFP chickens, while PWM-induced lymphocyte proliferation was similar between the two lines (*P* = 0.0677).

### Antibody concentrations in plasma

A significant interaction of sex × line (*P* = 0.0024) was observed for IgA antibody concentrations in plasma (Fig. 10B). Within both lines, roosters had lower IgA plasma concentrations than hens (HFP ♂ vs. ♀: *P* = 0.0001; LFP ♂ vs. ♀: *P* = 0.0298). However, roosters of the HFP line had lower IgA concentrations in plasma compared to roosters of the LFP line (*P* = 0.0062), whereas the IgA concentration in the plasma of hens did not differ between the two lines (*P* = 0.2246). Irrespective of line, roosters had a lower IgM concentration in plasma compared to hens (*P* < 0.0001).

### Summary of results

In summary, the results showed significant immunological differences between roosters and hens, with some sex effects only evident in one of the two investigated lines (Appendix 2). Furthermore, distinct immunological differences between HFP and LFP chickens were observed, with some line effects only evident in one sex (Appendix 3).

## Discussion

In the present study, substantial immunological differences between sexes, lines, and tissues (blood, spleen, cecal tonsils) were found with some effects specific to only one sex or line.

### Sex-differences in immune cell numbers and densities across lymphatic tissues

Roosters had higher total immune cell numbers compared to hens per spleen and cecal tonsil. In the spleen, this aligns with the larger absolute and relative organ mass in roosters. When normalized per gram of spleen, sex differences are less pronounced, indicating similar cell densities between sexes, and suggesting that the greater total immune cell reservoir in roosters is primarily due to organ mass. Honaker et al. (2024) did not observe sex differences in chickens at 91 days of age, suggesting that sex-related variation in relative spleen mas, and consequently potentially also in immune cell numbers, may become more apparent in adulthood. A different pattern emerged in the cecal tonsils. Despite smaller cecal tonsil mass, roosters exhibited a greater immune cell reservoir across most cell types, and sex differences persisted when normalized per gram, indicating higher immune cell density in the cecal tonsils of roosters compared to hens. By maintaining a high concentration of immune cells, the cecal tonsils might enable roosters to respond more quickly and effectively to local infections or antigens present in the digestive tract. Larger spleens and locally increased immune surveillance in specific mucosal tissues or lymphatic organs such as the cecal tonsils might enable avian males to compensate for possible testosterone-induced systemic immunosuppression (Trigunaite et al., 2015). To the best of our knowledge, no other studies have so far compared total immune cell numbers in the lymphoid tissues of roosters and hens. It was demonstrated that male broilers tend to have less cecal gut damage following exposure to *Eimeria tenella* than females (Boulton et al., 2018), and that more male embryos survive in ovo infections with *Eimeria tenella* (Jeffers and Wagenbach, 1969). This indicates a stronger resistance of roosters to coccidiosis. In contrast, Barbour et al. (1995) reported higher mortality in roosters in response to coccidiosis. Interestingly, female piglets were reported to synthesize more IgA in mesenteric lymph nodes, whereas male piglets synthesize more in cecal mucosa in response to probiotic supplementation, suggesting increased trafficking of plasma cells to the mucosal tissue in males (Christoforidou et al., 2019). Consistent with our findings, this suggests that roosters may have a higher potential of generating mucosal immune responses than hens.

### Sex differences in immune cell profiles

In our study, roosters had lower peripheral numbers and proportions of peripheral CD4^+^ cells, comprising mainly T helper cells, thus resulting in a lower CD4^+^/CD8α^+^ ratio. As this ratio is a marker for immune cell balance and competence (Lu et al., 2015; Mortada et al., 2021), these results suggest a lower capacity to regulate adaptive immune responses, including B cell-mediated responses at this age.

Lower B cell-mediated responses would align with the lower density and proportion of B cells, along with lower antibody concentration and B lymphocyte proliferation capacity observed in the present study. In contrast, Lyte et al. (2025) reported no difference in plasma IgY or cecal IgA concentration in younger chickens, but line-dependent differences in cecal IgA concentrations in female, but not in male, chickens of comparable age. Moreover, Christoforidou et al. (2019) showed that female piglets synthesized more IgA in mesenteric lymph nodes, whereas male piglets produced more in cecal mucosa in response to probiotic supplementation. These findings highlight that sex differences in antibody concentrations can be tissue-specific and underscore the importance of assessing antibody concentrations across multiple compartments.

Furthermore, roosters had a higher number and proportion of γδ T cells across all analyzed compartments. In chickens, γδ T cells represent a large fraction of T cell subsets involved in immune responses during infection (Berndt et al., 2006; Kano et al., 2009; Laursen et al., 2018; Pieper et al., 2011) with MHC-unrestricted cytotoxic functions (Fenzl et al., 2017). Altogether, these results suggest that roosters rely more on cytotoxic or innate immune mechanisms than on T-helper cell-mediated responses than hens. Hence, roosters may potentially be more effective to fight against intracellular bacteria but be more vulnerable to extracellular pathogens and infections that require strong antibody-mediated responses. In contrast, hens might focus on regulatory and antibody-based responses, suggesting a stronger adaptive immune response with a more effective defense against extracellular pathogens and response to vaccines. However, this assumption needs to be validated in further studies. These results match observations in humans and rodents, where females typically exhibit higher CD4^+^ frequencies and CD4^+^/CD8^+^ ratios (Amadori et al., 1995; Arsenović-Ranin et al., 2017; Grossman, 1985; Jentsch-Ullrich et al., 2005), higher B cell frequencies and enhanced B cell survival, faster maturation, and enhanced class switching compared to males (Abdullah et al., 2012; Aguirre-Gamboa et al., 2016; Fish, 2008; Lee et al., 1996; Schlinzig et al., 2017; Uppal et al., 2003), but lower γδ T cell (Michishita et al., 2011; Schultze-Florey et al., 2021; Singh et al., 2022) and natural killer cell numbers (Stefanski and Grüner, 2006).

The observation that some immune cell types are influenced by sex, while others remain unaffected, suggests that different immune cell populations are differentially regulated and/or more sensitive to modulating factors. These very likely might be a combination of genetics, tissue-specific microenvironments, and hormonal influences.

### Underlying mechanisms of sex differences in immunity

The mechanisms underlying sexual dimorphism in immunity are multifactorial, involving both differences in sex hormone concentrations and sex chromosomes (reviewed in Guerra-Silveira and Abad-Franch, 2013; Oertelt-Prigione, 2012).

Sex hormones exert immunoregulatory effects both directly, by acting on effector immune cells or immune organs through specific receptors, and indirectly, by modulating endocrine functions via their impact on the hypothalamus and pituitary gland (Grossman, 1985; Leitner et al., 1996; Yamamoto, 1985). Sex steroid receptors can be found on various lymphoid organs such as the thymus, bursa of Fabricius, and the spleen, as well as on various leukocytes, modulating their activation, lifespan, and functional response (Katayama et al., 2012; Shin et al., 2008; Sullivan and Wira, 1979). Estrogens or androgens are synthesized in both hens and roosters, but in different concentrations (Leitner et al., 1996; Younis and Ghoneim, 2022). Estradiol has been shown to enhance humoral immune responses and stimulate lymphocyte proliferation at physiological levels in poultry, whereas testosterone and dihydrotestosterone either had only weak stimulatory effects (Redig et al., 1985), no effect (Landsman et al., 2001; Leitner et al., 1996), or even suppressive effects, particularly on the bursa of Fabricius (Leitner et al., 1996; Novotny et al., 1983). In contrast, estradiol had no effect on bursal growth at low doses (Norton and Wira, 1977). The lower number and proportion of B cells, as well as a lower lymphocyte proliferation response to PWM and antibody concentration observed in roosters, may reflect the suppressive role of androgens on B cell development and function. Our results consistently showed higher numbers and proportions of γδ T cells in roosters, aligning with previous observations of an increase in γδ T cells in roosters, but not in hens, at the time of sexual maturation (Arstila and Lassila, 1993). Likely, testosterone treatment induced a strong increase in the regulatory T cell population and enhanced their differentiation and proliferation in humans (Walecki et al., 2015). This suggests that testosterone plays a role in T cell subset dynamics.

Sex is genetically determined by the differential inheritance of sex chromosomes, which influences immune-related gene expression that might contribute to the observed differences in immune cell numbers between roosters (ZZ) and hens (ZW). Indeed, although interferone-α and -β gene clusters are located on the Z chromosome in chickens (Ankra-Badu and Aggrey, 2005), bone marrow-derived macrophages from the heterogametic females show higher expression of IFN-inducible genes (Garcia-Morales et al., 2015). Similarly, Z-linked genetic regions associated with resistance to Marek’s Disease may help explain why females are often more susceptible to this disease than males (Heifetz et al., 2009; Lipkin et al., 2022).

Taken together, these findings suggest that both sex chromosome-linked gene expression and sex hormones likely contribute to the sexual dimorphism observed in our study.

### Line-differences in immune cell profiles

Immunological differences were also demonstrated between both lines. Overall, the HFP line consistently had lower immune cell numbers in both blood and spleen, suggesting genetic differences in immune cell availability or mobilization. In contrast, the cecal tonsils revealed more pronounced sex-dependent line effects, particularly in CD4^+^ cells and their subsets. This suggests a strong interaction between sex and genetic background in gut-associated immune cell profiles, and tissue-specific regulation of immune cell populations.

The observed lower numbers of respective immune cells in hens of the HFP line compared to hens of the LFP line were specifically CD4^+^ cells, CD25^dim^CD4^+^ cells, and CD25^high^CD4^+^ cells, which has already been discussed in Hofmann et al. (2025). However, the extended analysis of the present study revealed that differences in the number of these respective immune cell types between both lines are sex-specific, occurring exclusively in hens and localized specifically to the cecal tonsils. Notably, the incidence of feather pecking is higher in hens compared to roosters (Hughes, 1973). In line with this, Mindus et al. (2021) demonstrated that supplementation with *Lactobacillus rhamnosus* attenuates the stress-induced increase in severe feather pecking, an effect that was accompanied by an increased proportion of regulatory T cells in the tonsils. This finding further supports the hypothesis that T cell- mediated immunity, and potentially an altered immune balance originating in the gut, may be one contributing factor to feather pecking.

The fact that sex-dependent line effects are observed in tissue, but not in blood, could be due to tissue-specific immune activity, local microenvironments and hormonal regulation. Tissues, such as the cecal tonsils or the spleen, have specialized immune functions and are more directly involved in immune defense, which makes these differences more visible. Blood, on the other hand, is more likely to show the general state of the immune system, where such specific effects may be absent or weak.

## Conclusion

In summary, this study clearly demonstrates sex-specific differences in immune cell populations within both the systemic and the gut-associated immune system of hens and roosters. These findings underscore the need for more detailed investigations into the impact of sex on immune responses, particularly within the gut. The consequences of sexual dimorphism for immunity should be considered in the poultry industry, especially in the context of vaccination strategies, disease management, and selective breeding programs. Moreover, recognizing these differences is essential in research to prevent misinterpretations of immune competence and to ensure accurate assessment of immune traits across sexes. It is important to emphasize that differences in certain immune cell populations within the cecal tonsils between HFP and LFP chickens were observed only in hens, but not in roosters. As feather pecking behavior is particularly found in female birds, this finding supports the hypothesis that an altered immune balance, potentially originating from the gut, might be one contributing factor to feather pecking.

## CRediT authorship contribution statement

**Tanja Hofmann:** Writing – original draft, Visualization, Validation, Project administration, Methodology, Investigation, Formal analysis, Data curation, Conceptualization. **Volker Stefanski:** Writing – review & editing, Resources, Conceptualization. **Werner Bessei:** Writing – review & editing, Conceptualization. **Sonja Schmucker:** Writing – review & editing, Visualization, Validation, Supervision, Methodology, Data curation, Conceptualization.

## Disclosures

The authors declare that they have no known competing financial interests or personal relationships that could have appeared to influence the work reported in this paper.

## Appendix Supplementary materials

Image, application 1

## Footnotes

Supplementary material associated with this article can be found, in the online version, at doi:10.1016/j.psj.2026.106880.
