# Supplementary material for: Hens and roosters of two distinct lines differ in immune cell profiles
Source: Poult Sci. 2026 Mar 27;105(7):106880. doi: 10.1016/j.psj.2026.106880 (PMC13087782; doi:10.1016/j.psj.2026.106880)
Supplement: Supplementary file 1 [file mmc1.pdf]

Appendix 1: Gating strategy for the discrimination of immune cells in whole blood, spleen and cecal tonsils.

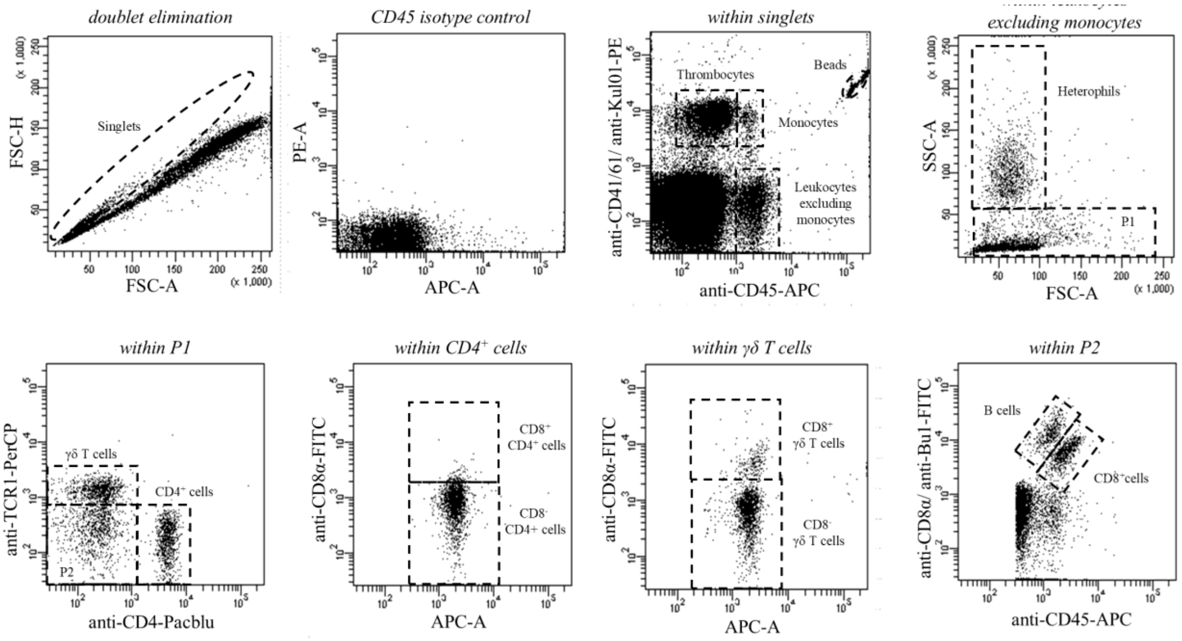

Figure 1: Gating strategy for the discrimination of immune cells in whole blood. Doublets were by plotting FSC-H versus FSC-A. An isotype control was applied to determine non-specific binding of total leukocytes. Single cells were classified into thrombocytes ($CD45^{dim}/CD41/61^{+}$), monocytes ($CD45^{+}/Kul01^{+}$), and leukocytes excluding monocytes ($CD45^{+}/Kul01^{-}$). The $CD45^{+}/Kul01^{-}$ population was further assessed by FSC/SSC to identify and exclude heterophils ($CD45^{+}/FSC^{low}/SSC^{dim/high}$). The remaining leukocytes (P1) were subsequently subdivided based on TCR1 and CD4 expression into γδ T cells ($CD45^{+}/TCR\gamma\delta^{+}$) and $CD4^{+}$ T cells ($CD4^{+}/TCR\gamma\delta^{-}$). Each subset was further distinguished by CD8α expression into $CD8\alpha^{+}$ and $CD8\alpha^{-}$ cells. All $CD4^{-}/TCR\gamma\delta^{-}$ leukocytes (P2) were displayed in a FITC/APC dot plot to simultaneously identify B cells ($Bu1\text{-}FITC^{high}/CD45\text{-}APC^{dim}$) and $CD8\alpha^{+}$ cells ($CD8\alpha\text{-}FITC^{dim}/CD45\text{-}APC^{high}$). Shown are representative dot plots of a rooster.

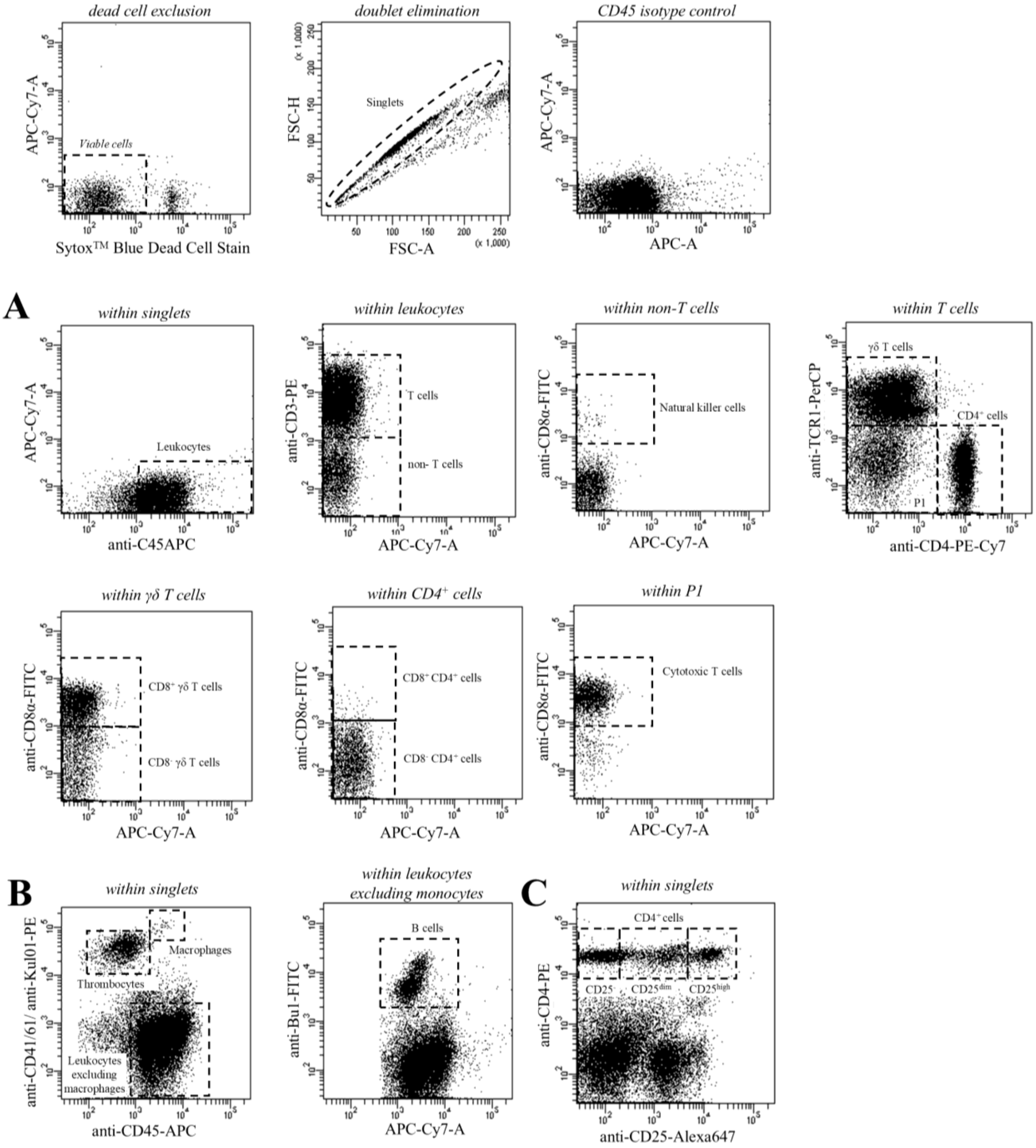

Figure 2: Gating strategy for the discrimination of splenic immune cells. Splenocytes were stained with three antibody panels (A, B, and C). An isotype control was used to define non-specific binding of leukocytes. In all staining protocols, dead cells and doublets were excluded as an initial step to ensure accurate analysis. Using Mix A, single cells were gated based on CD45 expression to identify total leukocytes ($CD45^{high}$). These were further separated into $CD3^{-}$ non-T cells and $CD3^{+}$ T cells. Within the non- T cell population, natural killer cells were identified ($CD45^{+}/CD3^{-}/CD8\alpha^{+}$). T cells were subdivided into γδ T cells ($CD45^{+}/CD3^{+}/TCR\gamma\delta^{+}/CD4^{-}$), CD4+ cells ($CD45^{+}/CD3^{+}/TCR\gamma\delta^{-}/CD4^{+}$), and cytotoxic T cells ($CD45^{+}/CD3^{+}/TCR\gamma\delta^{-}/CD4^{-}/CD8\alpha^{+}$). Both, $CD4^{+}$ cells and γδ T cells, were further classified as $CD8\alpha^{+}$ and $CD8\alpha^{-}$. Using Mix B, single cells were classified into thrombocytes (CD45dim/CD41/61+), macrophages (CD45+/Kul01+), and leukocytes excluding macrophages ($CD45^{+}/Kul01^{-}$), from which B cells were subsequently identified ($CD45^{+}/Kul01^{-}/Bu1^{+}$). Using Mix C, $CD4^{+}$ cells were further divided into $CD25^{-}$, $CD25^{dim}$, and $CD25^{high}$ subsets. A fluorescence minus one (FMO) control was used to accurately define CD25 expression levels.

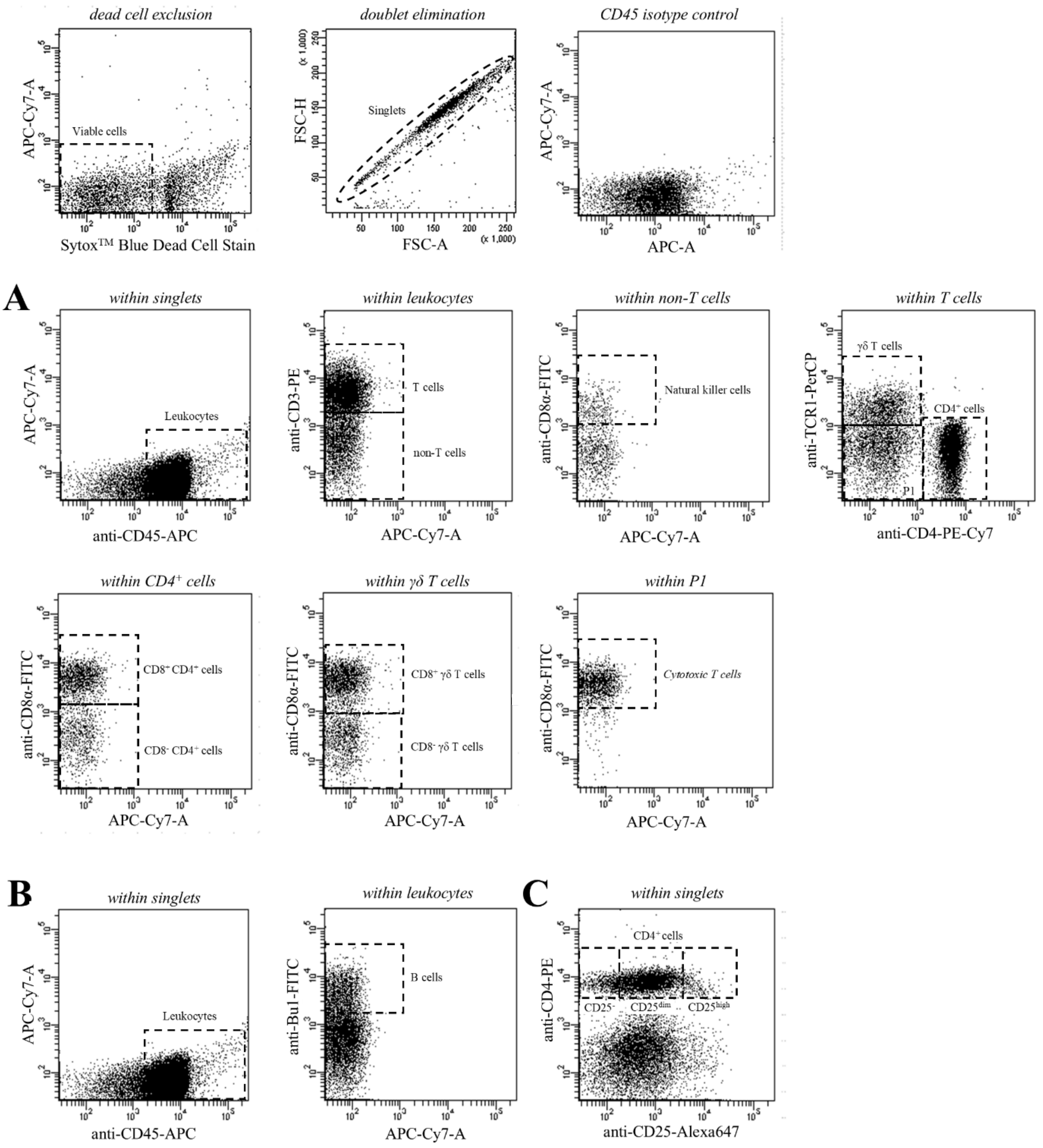

Figure 3: Gating strategy for the discrimination of immune cells in the cecal tonsils. Cell suspension from cecal tonsils were stained using three antibody panels (A, B, C). An isotype control was used to define non-specific binding of leukocytes. In all staining protocols, dead cells and doublets were excluded as an initial step to ensure accurate analysis. Using Mix A, single cells were gated based on CD45 expression to identify total leukocytes ($CD45^+$). These were further divided into $CD3^-$ non-T cells and $CD3^+$ T cells. Within the non-T cell population, natural killer cells were identified ($CD45^+/CD3^-/CD8\alpha^+$). T cells were subdivided into γδ T cells ($CD45^+/CD3^+/TCR\gamma\delta^+/CD4^-$), $CD4^+$ cells ($CD45^+/CD3^+/TCR\gamma\delta^-/CD4^+$), and cytotoxic T cells ($CD45^+/CD3^+/TCR\gamma\delta^-/CD4^-/CD8\alpha^+$). Both $CD4^+$ cells and γδ T cells were further classified into $CD8\alpha^+$ and $CD8\alpha^-$. Using Mix B, leukocytes within the single-cell population were identified ($CD45^+$), and B cells were subsequently characterized ($CD45^+/Bu1^+$). Using Mix C, $CD4^+$ cells were further divided into $CD25^-$, $CD25^{dim}$, and $CD25^{high}$ subsets. A fluorescence minus one (FMO) control was used to accurately define CD25 expression levels.

Appendix 2: Summary of total sex effects (line × sex: HFP ♂ vs. HFP ♀, LFP ♂ vs. LFP ♀; sex: ♂ vs. ♀) in blood, spleen and cecal tonsils.

	**Blood**		**Spleen**			**Cecal tonsils**		
**Immune cell type**	*#/µL*	*% of leukocytes*	*#/g*	*#/spleen*	*% of leukocytes*	*#/g*	*#/CT*	*% of leukocytes*
**Leukocytes**	ns	x	HFP: ♂ < ♀	♂ > ♀	x	♂ > ♀	♂ > ♀	x
**Thrombocytes**	♂ < ♀	x	♂ > ♀	♂ > ♀	x	x	x	x
**Monocytes/Macrophages**	ns	ns	ns	♂ > ♀	ns	x	x	x
**Heterophils**	ns	ns	x	x	x	x	x	x
**Natural Killer cells**	x	x	ns	♂ > ♀	ns	HFP: ♂ > ♀	ns	♂ < ♀
**$CD4^+$ cells**	♂ < ♀	♂ < ♀	ns	♂ > ♀	ns	♂ > ♀	♂ > ♀	♂ > ♀
**$CD8\alpha^+$ cells**	ns	ns	x	x	x	x	x	x
**Cytotoxic T cells**	x	x	HFP: ♂ < ♀	ns	ns	♂ > ♀	ns	♂ < ♀
**γδ T cells**	♂ > ♀	♂ > ♀	LFP: ♂ > ♀	♂ > ♀	♂ > ♀	♂ > ♀	♂ > ♀	LFP: ♂ > ♀
**Total T cells**	x	x	HFP: ♂ < ♀	♂ > ♀	♂ > ♀	♂ > ♀	♂ > ♀	♂ > ♀
**B cells**	♂ < ♀	♂ < ♀	♂ < ♀	ns	♂ < ♀	♂ > ♀	♂ > ♀	ns
**$CD25^-$ $CD4^+$ cells**	x	x	HFP: ♂ < ♀	ns	HFP: ♂ < ♀	♂ > ♀	♂ > ♀	ns
**$CD25^{dim}$ $CD4^+$ cells**	x	x	♂ > ♀	♂ > ♀	♂ > ♀	♂ > ♀	♂ > ♀	♂ > ♀
**$CD25^{high}$ $CD4^+$ cells**	x	x	ns	♂ > ♀	ns	♂ > ♀	♂ > ♀	♂ < ♀

ns = not significant; x = not analysed

Appendix 3: Summary of total line effects (line × sex: ♂ HFP vs. ♂ LFP; ♀ HFP vs. ♀ LFP; line: HFP vs. LFP) in blood, spleen and cecal tonsils.

	**Blood**		**Spleen**			**Cecal tonsils**		
**Immune cell type**	*#/µL*	*% of leukocytes*	*#/g*	*#/spleen*	*% of leukocytes*	*#/g*	*#/CT*	*% of leukocytes*
**Leukocytes**	HFP < LFP	x	♂: HFP < LFP	HFP < LFP	x	♀: HFP < LFP	ns	x
**Thrombocytes**	HFP < LFP	x	ns	HFP < LFP	x	x	x	x
**Monocytes/Macrophages**	ns	HFP > LFP	ns	HFP < LFP	HFP > LFP	x	x	x
**Heterophils**	ns	HFP > LFP	x	x	x	x	x	x
**Natural Killer cells**	x	x	ns	HFP < LFP	ns	♂: HFP > LFP	ns	ns
**$CD4^+$ cells**	HFP < LFP	HFP < LFP	HFP < LFP	HFP < LFP	HFP < LFP	♂: HFP > LFP<br>♀: HFP < LFP	♀: HFP < LFP	♂: HFP > LFP<br>♀: HFP < LFP
**$CD8\alpha^+$ cells**	HFP < LFP	HFP < LFP	x	x	x	x	x	x
**Cytotoxic T cells**	x	x	ns	HFP < LFP	HFP > LFP	ns	ns	HFP > LFP
**γδ T cells**	HFP < LFP	HFP < LFP	♂: HFP < LFP<br>♀: HFP > LFP	♂: HFP < LFP	♀: HFP > LFP	ns	HFP < LFP	♂: HFP < LFP
**Total T cells**	ns	x	♂: HFP < LFP	HFP < LFP	HFP > LFP	ns	ns	HFP > LFP
**B cells**	HFP < LFP	HFP < LFP	HFP < LFP	HFP < LFP	HFP < LFP	HFP < LFP	HFP < LFP	HFP < LFP
**$CD25^-$ $CD4^+$ cells**	x	x	HFP < LFP	HFP < LFP	♂: HFP < LFP	♂: HFP > LFP<br>♀: HFP < LFP	♀: HFP < LFP	ns
**$CD25^{dim}$ $CD4^+$ cells**	x	x	HFP < LFP	HFP < LFP	♂: HFP > LFP	♂: HFP > LFP<br>♀: HFP <LFP	♀: HFP < LFP	ns
**$CD25^{high}$ $CD4^+$ cells**	x	x	HFP < LFP	HFP < LFP	ns	♀: HFP < LFP	HFP < LFP	♂: HFP < LFP

ns = not significant; x = not analysed
